# Changes on the Structural and Physicochemical Properties of Conjugates Prepared by the Maillard Reaction of Black Bean Protein Isolates and Glucose with Ultrasound Pretreatment

**DOI:** 10.3390/polym11050848

**Published:** 2019-05-10

**Authors:** Hua Jin, Qingshan Zhao, Haiying Feng, Yuxin Wang, Jubing Wang, Yanlong Liu, Dong Han, Jing Xu

**Affiliations:** 1 College of Science, Northeast Agricultural University, Harbin 150030, Heilongjiang, China; jinhua@neau.edu.cn (H.J.); 18745724605@163.com (H.F.); wyx854994@163.com (Y.W.); 15663431982@163.com (J.W.); liuyanlonghyy@163.com (Y.L.); hanhahahhh@163.com (D.H.); 2 Laboratory Management Office, Northeast Agricultural University, Harbin 150030, Heilongjiang, China; zhaoqingshan@neau.edu.cn

**Keywords:** black bean protein isolate, glucose, Maillard reaction, ultrasound pretreatment

## Abstract

The conjugates of black bean protein isolate (BBPI) and glucose (G) were prepared via the wet heating Maillard reaction with ultrasound pretreatment. The physicochemical properties of UBBPI-G conjugates prepared by ultrasound pretreatment Maillard reaction had been compared with classical Maillard reaction (BBPI-G). The reaction rate between BBPI and glucose was speeded up by ultrasound pretreatment. A degree of glycation (DG) of 20.49 was achieved by 2 h treatment for UBBPI-G, whereas 5 h was required using the classical heating. SDS-PAGE patterns revealed that the BBPI-G conjugates with higher molecular weight were formed after glycosylation. The results of secondary structure analysis suggested that the α-helix and β-sheet content of UBBPI-G were lower than that of BBPI-G. In addition, UBBPI-G conjugates had exhibited bathochromic shift compared with BBPI by fluorescence spectroscopy analysis. Finally, UBBPI-G achieved higher level of surface hydrophobicity, solubility, emulsification property and antioxidant activity than BBPI and BBPI-G (classical Maillard reaction).

## 1. Introduction

Black soybean is planted and consumed in various regions of the world, as they are nutritionally rich in biologically active compounds, such as proteins, essential amino acids, anthocyanins, isoflavones and polyunsaturated fatty acids so on [1]. Furthermore, the black soybean has higher protein content than other kinds of soybean and contains a favorable balance of amino acids [2]. Therefore, black soybean is an excellent source of protein for extraction and modification. Black bean protein isolate, due to its good solubility, emulsifying attributes and antioxidant activity, has exhibited remarkable potential of application in the food industry [2,3]. Moreover, these functional properties can be improved by appropriate modifications, including physical, chemical and enzymatic treatments [2,3,4]. However, up to now, most of the researches on black soybean have focused on the anthocyanin and phenolics in seed coat [1], while the protein ingredient has not received enough attention.

Recently, the conjugate of protein and sugar produced by Maillard-type reaction has attracted much attention because of its commendable functional properties as well as the antioxidant activity and emulsifying properties [5]. Mu et al. [6] reported that the solubility of soy protein isolates-acacia gum conjugates was significantly (*p* < 0.05) higher than that of unreacted soy protein isolates-acacia gum mixtures and soy protein isolates at the same pH values. Furthermore, there are also some researches having shown that the conjugates with enhanced emulsifying properties [7], strong antimicrobial ability [8], and increased heat stability [9] may be formed during the Maillard reaction. However, protein-sugar graft reaction is time-consuming through classical heating [10]. Thus, a more rapid and effective method is needed for improving the classical Maillard-type reaction in order to produce more protein-sugar conjugates. 

Ultrasound with the frequency 2 × 10^4^–1 × 10^9^ Hz can produce high shear and mechanical energy induced by the cavitation phenomenon. Ultrasonic technology shows many advantages like simple operation, low cost, short treatment time and non-toxic side effects. Therefore, it has become popular to modify the structures and properties of proteins [5]. Moreover, application of an ultrasonic technique can also greatly decrease both analysis and processing times in the food industry [11]. Recent study [12] has found that the Maillard reaction by using the ultrasonic assisted treatment could be greatly accelerated, because the more free amino groups and energy were supplied for the graft process by the ultrasound treatment, which facilitated the graft process of the protein and sugar. Similarly, Mu et al. [11] found that after wet-heating at 80 °C for 32 h, the degree of graft was 34.08% between soy protein isolate and gum acacia glycosylation, whereas a DG of 34.11% was achieved at a much shorter reaction time of 60 min with the assistance of ultrasound. Meanwhile, grafted protein showed higher level of the physicochemical and functional property by ultrasonic assisted treatment than the classical heating Maillard and native protein. On the other hand, the emulsifying properties of the glycosylated conjugates obtained by ultrasound-assisted wet heating Maillard reaction were significantly improved [5]. However, the modification of BBPI via such ultrasound-assisted Maillard reaction remains poorly studied. These researches could supply more information for broadening black soybean protein utilization as value-added conjugate apply in the food industry.

Herein, BBPI was modified via the classical Maillard reaction (BBPI-G) and ultrasound-assisted Maillard reaction (UBBPI-G), respectively, to compare the differences of the conjugates obtained by two modified methods on structural and functional properties. The electrophoretic property, secondary structure (FTIR spectroscopy), tertiary structure (fluorescence spectroscopy), surface hydrophobicity (*H*_0_), solubility, emulsifying properties, antioxidant activity of samples were investigated. We also analysed the properties changes of BBPI-G and UBBPI-G at different Maillard reaction time (1–6 h) to determine the impact trend. 

## 2. Materials and Methods

### 2.1. Materials

Black soybeans (*G. max* L. merr) were supplied from Hei Long Jiang Agriculture Company Limited (Harbin, Hei Long Jiang, China). Glucose was from Baolingbao Biology Company (Yucheng, Shangdong, China). Sodium dodecyl sulphate (SDS) and Ferrozine were purchased from Sigma-Aldrich Co. (St. Louis, MO, USA). All other chemicals were of analytical grade.

### 2.2. Preparation of Ultrasound Pretreatment BBPI

The preparation of BBPI followed the method reported by Jiang, et al. [2]. Phosphate buffer (0.1 M, pH 7.0) was added to the BBPI powder and the mixture solution was stirred for 2 h at an ambient temperature (20 °C). A 5 mg/mL total protein concentration solution was obtained. This solution was treated by an ultrasound processor (NingBo Scientz Biotechnology Co. Ltd., Ningbo, Zhejiang, China) under the ultrasound power and frequency of 150 w and 20 kHz for 30 min (pulse duration of on-time 2 s and off-time 2 s). During the ultrasonic process, an ice-water bath was used to control the reaction temperature at 20 °C. After ultrasonic pretreatment, we got the black soybean protein of ultrasound pretreatment (UBBPI).

### 2.3. Preparation of BBPI-G and UBBPI-G Conjugates

BBPI-G and UBBPI-G conjugates were respectively mixed with glucose at the same mass ratio (*w*/*w*) of protein (5 mg/mL), glucose = 2:1 in phosphate buffer (0.1 M, pH 7.0), which were thoroughly mixed. Then, the solution was incubated at 80 °C for different times (1–6 h). After graft process, all preparations were ended by cooled down to the ambient temperature, and the corresponding lyophilized samples were named as UBBPI-G or BBPI-G conjugates.

### 2.4. Degree of Glycation (DG)

The slightly modified *o*-phthaldialdehyde (OPA) assay was used to measure the free amino groups [13]. 1 mL OPA (40 mg) methanol solution, 25 mL Na_2_B_4_O_7_ solution (100 mM), 2.5 mL 20% (*w*/*v*) SDS and 100 μL β-mercaptoethanol were mixed and diluted to 50 mL by distilled water to obtain the OPA reagent. Thus, 4 mL OPA reagent was mixed with 200 μL protein samples (5 mg/mL), and incubated for 2 min at 35 °C. Using the deionized water as blank, the absorbance was measured at 340 nm. According to the standard curve constructed using 0.25–2 mM L-lysine, the free amino groups could be calculated.

DG (%) = (*A*_0_ − *A*_t_)/*A*_0_ × 100%

where *A*_0_ is the absorbance of the sample before Maillard reaction, and *A*_t_ is its level after Maillard reaction for t h.

### 2.5. Browning Value

The browning value was determined as previously reported [14] with some modification: 0.1% (*w*/*v*) SDS was used to dilute protein samples to 0.2% (*w*/*v*) as blank. The absorbance at 420 nm was detected to evaluate browning value.

### 2.6. Sodium Dodecylsulfate Polyacrylamide Gel Electrophoresis (SDS-PAGE)

SDS-PAGE was carried out on a discontinuous-buffer system [15] on 12% (*v*/*v*) separating gel and 5% (*v*/*v*) stacking gel. The gels were stained with Coomassie Blue after the run.

### 2.7. Fluorescence Spectroscopy

The F-4500 Fluor photometer (Hitachi, Tokyo, Japan) was used to obtain the fluorescence spectra. The protein solution (0.2 mg/mL) was dissolved with phosphate buffer (pH 7.0, 10 mM). Using 295 nm as excitation wavelength, the emission spectra were recorded from 300 to 440 nm with a constant slit of 5 nm [16].

### 2.8. Fourier Transform Infrared (FTIR) Spectroscopy

Infrared spectra were measured by a Bruker Vertex 70 FTIR spectrometer (Bruker Optics, Ettlingen, Baden-Württemberg, Germany) from 400 to 4000 cm^−1^ with a resolution of 4 cm^−1^ in 64 scans. The secondary structure content of protein was calculated via the software “Peakfit Version 4.12” (SPSS Inc., Chicago, IL, USA) and “Gaussian peak fitting” (SPSS Inc., Chicago, IL, USA) algorithm [17].

### 2.9. Surface Hydrophobicity (H_0_)

Surface hydrophobicity (*H*_0_) was determined according to Haskard and Li-Chan [18]. 1,8-anilinonaphthalenesulfonate (ANS) was the fluorescence probe used to determine the surface hydrophobicity (*H*_0_) values of proteins. The protein solutions (0.04–0.4 mg/mL) were prepared in phosphate buffer (10 mM, pH 7.0). Then 4 mL of the above solutions were mixed with 40 μL ANS (8.0 mmol/L). The fluorescence intensity (FI) was measured wavelength of 468 nm (emission) using an excitation at 390 nm. The index of surface hydrophobicity (*H*_0_) was obtained from a plot of initial slope of the FI versus protein concentration (mg/mL).

### 2.10. Solubility

A slightly modified method of protein solubility measurement was used [19]. The protein solution (5 mg/mL), in phosphate buffer (10 mM, pH 7.0), was centrifuged at 12,000× *g* for 20 min. The protein content in the supernatant was determined by Coomassie blue method and the standard curve was constructed using bovine serum albumin.

### 2.11. Emulsifying Property

To assess the emulsifying property, the emulsifying activity index (EAI) and emulsifying stability index (ESI) were determined [20]. For emulsion formation, the protein solution (2 mg/mL) and soybean oil were homogenized at 3:1 (*v*/*v*) using the homogenizer (AE300L-H; Shanghai Angni Instruments Co., Shanghai, China). After the homogenization, emulsion (50 μL) was sucked from the bottom at 0 and 10 min, and then diluted with 0.1% (*w*/*v*) SDS solution in 1:100 (*v/v*). The measurement of absorbance was performed at 500 nm.

EAI (m^2^/g) = 2*T* × *A*_0_ × *N* × 10^−4^/ϕ*LC*

ESI(min) = *A*_0_ × *t*/(*A*_0_ − *A*_10_)

where *T* is 2.303, *A*_0_ and *A*_10_ are the absorbance at 0 and 10 min. *N* is dilution factor (100), ϕ is the oil volume fraction (0.25), *L* is path length of cuvette (1 cm), and *C* is protein concentration (g/mL).

### 2.12. Evaluation of Antioxidant Activity

#### 2.12.1. Iron Chelating Capacity

Iron chelating capacity of BBPI, UBBPI and UBBPI-G were evaluated by the method of Dinis et al. [21]. Using the distilled water as control, the absorbance value of the protein samples (*A*) and the control (*A*_0_) were measured at 562 nm.

Iron chelating capacity (%) = (1−*A*/*A*_0_) × 100%

#### 2.12.2. Reducing Power

Reduction capacity of BBPI, UBBPI and UBBPI-G was evaluated using the method of Oyaizu [22]. 1 mL of the BBPI, BBPI-G or UPPBI-G solution (5 mg/mL) at pH 6.6 adjusted by sodium phosphate buffer was blended with 1.0 mL of potassium ferricyanide (1%). The mixture was kept at 50 °C for 20 min, and then cooled to room temperature. 1.0 mL of trichloroacetic acid (10%) was added to above mixture, which was centrifuged next. The supernatant was 2-fold diluted using distilled water, and then 400 μL FeCl_3_ 0.1% was added. After dispersing and standing for 10 min, the absorbance was measured at 700 nm to assess the reducing power.

#### 2.12.3. Hydroxyl Radical Scavenging Rate

Hydroxyl radical scavenging rate of BBPI, UBBPI and UBBPI-G was evaluated by the method of Amarowicz et al. [23], using distilled water as blank. The distilled water instead of salicylic acid ethanol solution was taken as control. The absorbance at 510 nm was measured.

Hydroxyl radical scavenging rate (%) = [1 − (*A*_sample_ − *A*_control_)/*A*_blank_] × 100%

### 2.13. Statistical Analysis

All the experiments were repeated 3 times, and the results are given as means ±standard deviations. The analysis of significant differences (*p* < 0.05) was performed through Duncan’s multiple range test of SPSS (20.0) software (New York, NY, USA). 

## 3. Results and Discussion

### 3.1. Effect of Ultrasound Pretreatment on BBPI-G Grafting Reaction

**Figure 1:**
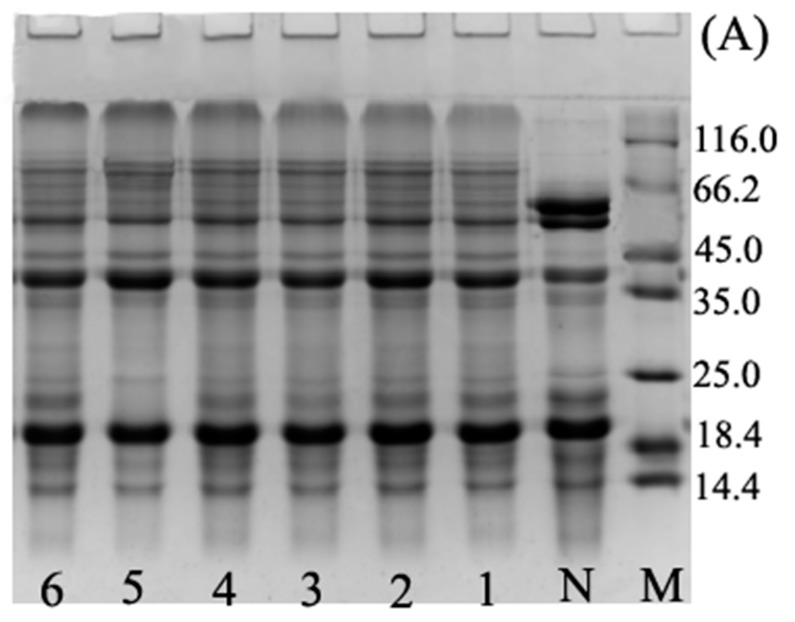

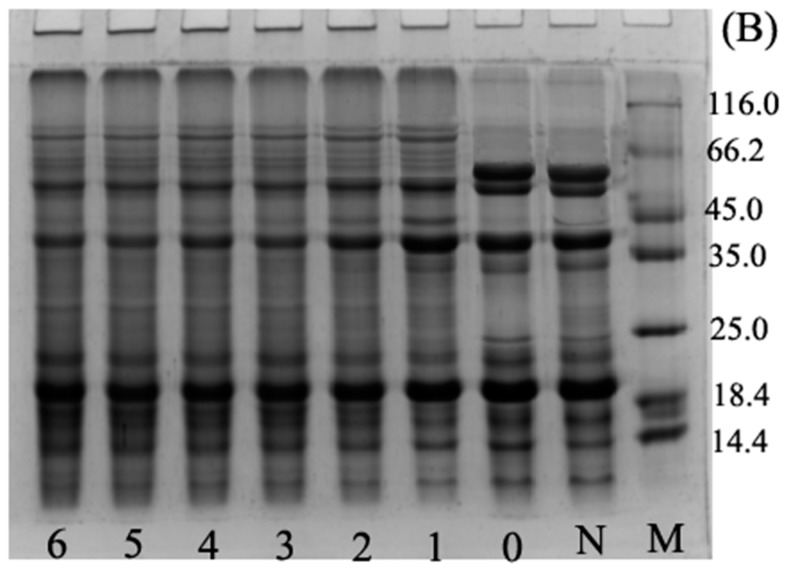
SDS-PAGE profiles of BBPI by wet heating (**A**) and ultrasound treatment Maillard reaction (**B**). Lane M: maker, lane N: native BBPI, lane 0: BBPI of ultrasound treatment, lanes 1–6: wet Maillard reaction (**A**) and ultrasound treatment Maillard reaction (**B**) for 1–6 h, respectively. The concentration of protein solution in each lane of the gels was 5 mg/mL.

The SDS-PAGE analysis reported in Figure 1 indicated that the ultrasound pre-treatment (Figure 1B, lane 0) did not induce any change in the protein pattern in comparison with BBPI (Figure 1B, lane N). This observation confirmed that there were no major changes in protein electrophoresis profiles for UBBPI samples, which was similar to the results of Jiang et al. [2]. We could see that for both BBPI-G (Figure 1A) and UBBPI-G (Figure 1B) the glucose molecules were combined with BBPI molecules, which could be confirmed by the characteristic new slower migrating bands in Figure 1. This suggested that high molecular weight protein-sugar conjugates were indeed generated. The result agreed well with previous research of soy protein isolate-glucose conjugates [6]. In addition, the ultrasound pretreatment induces a faster appearance of the high molecular weight components (Figure 1B) than wet Maillard reaction (Figure 1A). This phenomenon was similar to the SDS-PAGE patterns of peanut protein-maltodextrin conjugates produced by ultrasound-assisted wet heating Maillard reaction, which showed that ultrasound treatment could promote Maillard reaction and make the occurrence of protein glycation more readily [12]. Therefore, from these observations, it could be inferred that UBBPI-G should obtain higher DG compared with BBPI-G in the same time. 

The changes in DG and browning values of BBPI and glucose conducted by classical heating and ultrasound-assisted pretreatment are shown in Table 1. It was obvious that ultrasound-assisted Maillard reaction required less time to reach the same DG than the classical Maillard reaction. For example, a DG of 20.49% was obtained for UBBPI-G samples by 2 h, whereas a DG of 20.60% was obtained by classical heating after a much longer time of 5 h. This result was similar to that of previous study [12], and indicated that the Maillard reaction was effectively enhanced by ultrasound pretreatment. Because ultrasound could improve the rate of the heat and mass transport processes, provide good mixing and develop the graft reaction, it was considered to supply a sonocatalysis effect [12,24,25]. Moreover, ultrasound cavitation could speed up the protein molecules motion, rearrange and unfold the protein molecules, causing the protein secondary and tertiary structural changes [17]. These structural changes could induce the exposition in BBPI of more reactive free amino groups for the graft process [5,11]. 

Generally, the Maillard reaction is divided into three stages: initial, intermediate, and advanced. In the initial stage of Maillard reaction, the products formed via a condensation of carbonyl groups with amino group usually did not lead to an absorbance in the visible spectrum. In contrast, a melanoidin compounds with the maximum absorbance at 420 nm can appear in the latter two stages of Maillard reaction [26]. Therefore, the browning values became higher when the graft reaction time was extended (Table 1). However, compared with the BBPI-G conjugates, the UBBPI-G conjugates showed smaller browning values under the similar grafting degree, which meant that the ultrasonic treatment reduced browning intensity of the Maillard reaction. This phenomenon might suggest that ultrasonication could prevent the polymerization of intermediate products to form melanoidins during graft process [27]. Similarly, the browning values of β-conglycinin and maltodextrin conjugates prepared by classical Maillard reaction were also much higher than those by ultrasound treatment at the same DG [28]. Hence, it can be seen that ultrasound treatment not only accelerates the graft reaction, but also reduces the brown colours, which is favour of industrial application.

### 3.2. Fourier Transform Infrared (FTIR) Spectroscopy

The secondary structure of BBPI-G and UBBPI-G conjugates was analysed by FTIR spectroscopy and the calculated value of each secondary structure component is shown in Table 2. The results indicated that the secondary structure of BBPI was changed after both types of Maillard reaction. In particular, the unordered structure content (β-turn + random coil) grew evidently (*p* < 0.05) following the glucose attachment, but the ordered structure content (α-helix + β-sheet) declined in an opposite fashion. During the graft process, the interaction between BBPI and glucose molecules could affect the hydrogen bonds and van der Waals forces, which maintained the stability of the secondary structure of proteins. Therefore, the secondary structure of protein molecules was changed as a study of Zhang et al. [5]. On the other hand, due to the heating treatment during the Maillard reactions, the heat denaturation of proteins cannot be excluded [29]. Kim et al. [30] found that the soybean glycinin showed obvious changes in their secondary structure after heating above 80 °C.

Compared to BBPI-G, e UBBPI-G conjugates lost more ordered secondary structure content and gained more unordered structure content. This result was ascribed to the pressure alterations and turbulence caused by ultrasonic treatment, leading to structural transformations [25]. Moreover, ultrasound could partially destroy the interaction force between protein molecules by facilitate the glycosylation of protein and sugar. Therefore, UBBPI-G conjugates had more effective changes in the structure distribution, which could cause better uniformity and flexibility conjugates compared with classical Maillard reaction. It was known that the functionality was well related to the structure [7,11]. Unordered structure had better flexibility than ordered structure, which was beneficial to the function of protein. Mu et al. [11] showed that the greater the flexibility of the protein molecules, the better the emulsification.

### 3.3. Fluorescence Spectroscopy Analysis

**Figure 2:**
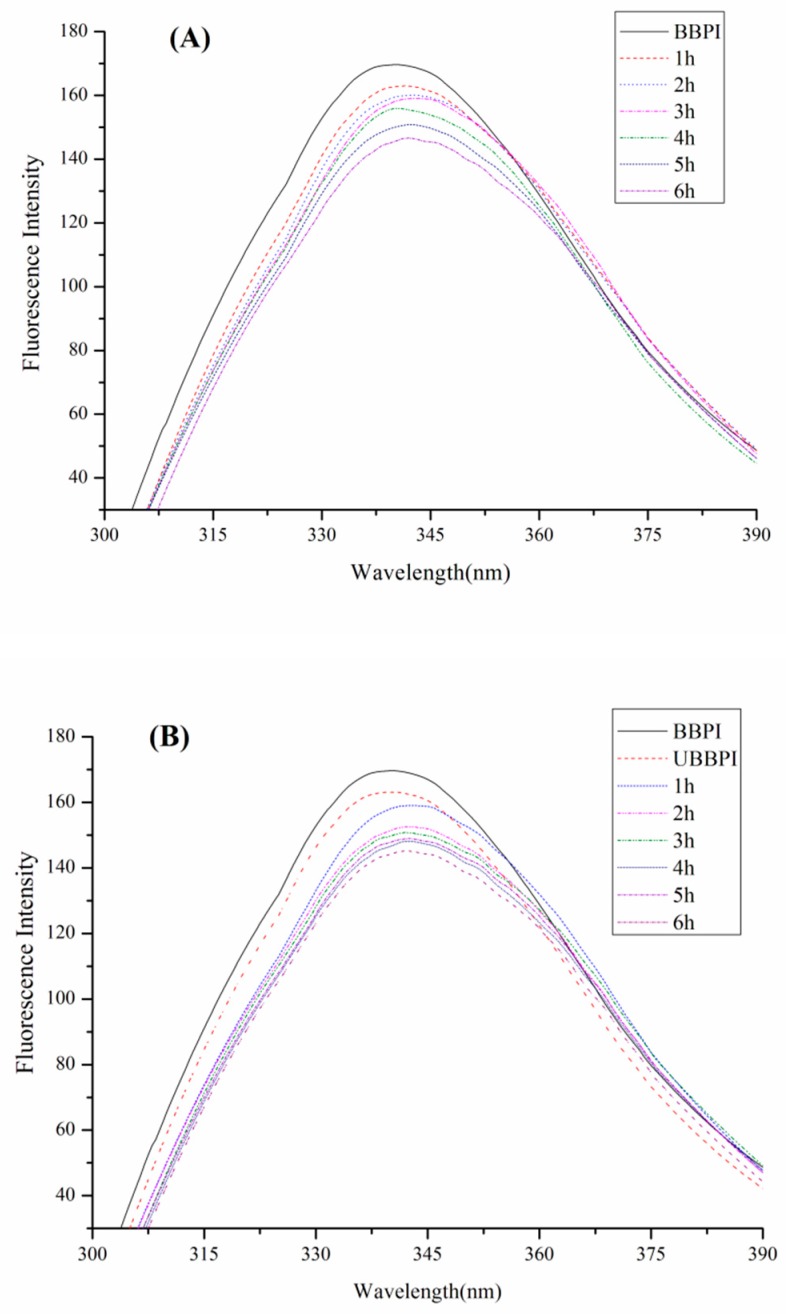
Fluorescence intensity of BBPI by wet heating (**A**) and ultrasound treatment Maillard reaction (**B**) respectively obtained at the indicated reaction time for 1–6 h.

Based on analysis of fluorescence spectroscopy, the microenvironment around tryptophan in proteins can be reflected to detect the changes of protein conformation [31]. The fluorescence spectroscopy of BBPI-G and UBBPI-G conjugates was shown in Figure 2. In comparison to BBPI, the λ_max_ of BBPI-G showed a bathochromic shift phenomenon, illustrating that the microenvironment of the tryptophan groups had become more polar (Figure 2A). Generally, the bathochromic shift phenomenon is known to appear with the increase of maximal fluorescence, but the fluorescence spectra decreased gradually here owing to the shielding effect of the hydrophilic sugar chain to the tryptophan residues. Similar results were also found in the previous work [25].

In addition, the UBBPI samples clearly exhibited lower FI after the ultrasound pretreatment (Figure 2B). Because this treatment could generate fluid mixing and shear forces by cavitation effects [32], the protein molecules were partly disrupted, and exposed more chromophores to the solvent, which led to the FI decrease [2,5]. Therefore, the tertiary structure of protein was changed after ultrasonic pretreatment. Based on this reason, the UBBPI-G conjugates showed even much lower fluorescence intensity than the BBPI-G conjugates. This result was in accord with the DG data in Table 1 showing that the UBBPI-G conjugates grafted with more glucose molecules, generating a stronger shielding effect in UBBPI-G conjugates obtained by ultrasound pretreatment Maillard reaction compared to classical heating. It possessed similar fluorescence features of the glycosylated products as given in previous reports [29], which might be related with the high degree of graft during prior Maillard reaction.

### 3.4. Surface Hydrophobicity (H_0_)

**Figure 3:**
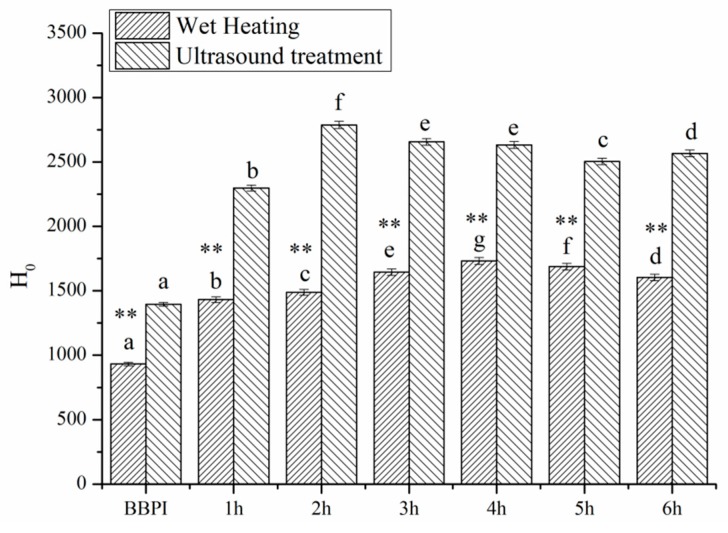
Surface hydrophobicity (*H*_0_) of BBPI by wet heating and ultrasound treatment Maillard reaction obtained for 1–6 h, respectively. Means with dissimilar lower-case letters indicate significant differences (*p* < 0.05), ** means in the same time *p* < 0.01, * means in the same time *p* < 0.05.

Surface hydrophobicity (*H*_0_) reflects the number of hydrophobic groups on the protein surface [33]. The effect of the classical wet heating and ultrasonic pretreatment Maillard reaction on the *H*_0_ values was shown in Figure 3. The result revealed that the *H*_0_ values of BBPI-G conjugates were much higher than those of BBPI. It was well known that most hydrophobic residues were buried in the interior of the compact globular region [34]. Hence, the fluorescence probe (ANS) binding to hydrophobic residues was disturbed, and the *H*_0_ value of BBPI was comparatively low [25]. According to previous study, Zhao et al. [27] showed that the *H*_0_ values of heated soy protein isolate was clearly higher than native soy protein isolate, meaning that more hydrophobic groups exposed on the protein surface after heating. This indicated that the surface hydrophobicity was influenced greatly by temperature factor. Based on this result, the conjugates glycosylated by β-conglycinin and dextran showed higher *H*_0_ value than native β-conglycinin, as expected [35]. A similar result was also reported by Wang et al. [29], who illustrated that the mung bean protein isolate (MBPI)-glucose (G) conjugates subjected to whether ultrasound treatment or classical heating both manifested the higher surface hydrophobicity than untreated MBPI, presumably resulting from aggregate dissociation or protein unfolding. In Figure 3, we also noticed that the *H*_0_ values of BBPI-G and UBBPI-G conjugates declined slightly with the ongoing increase in reaction time. This phenomenon may be due to the more hydrophilic glucose molecules linked to the proteins during the glycosylated reaction, which could be confirmed by the DG data in Table 1, partly covering the hydrophobic area of the proteins. 

Moreover, *H*_0_ values of UBBPI-G conjugates were even higher than those of BBPI-G, since the cavitation phenomenon and mechanical effect induced by ultrasonic treatment destroyed protein conformation and structure. Then, the hydrophobic residues buried in the protein interior were exposed towards the aqueous environment more effectively, which resulted in the enhancement of surface hydrophobicity [28].

### 3.5. Solubility

**Figure 4:**
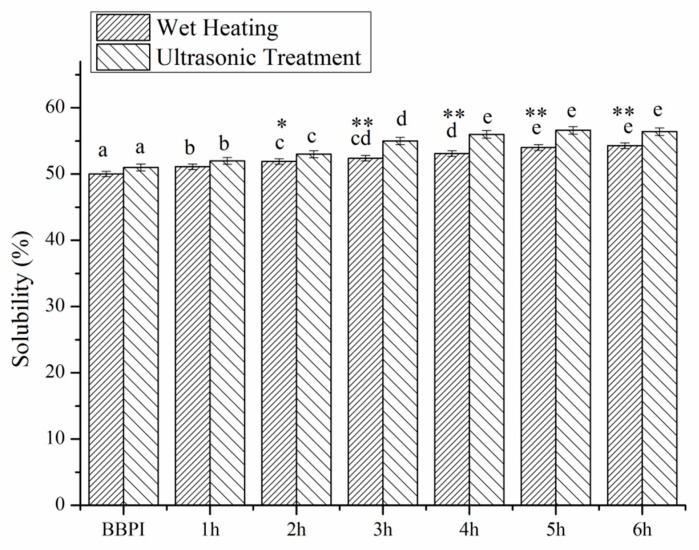
Solubility of BBPI by wet heating and ultrasound treatment Maillard reaction obtained after 1–6 h, respectively. Means with dissimilar lower-case letters indicate significant differences (*p* < 0.05), ** means in the same time *p* < 0.01, * means in the same time *p* < 0.05.

Solubility is an important physicochemical property of proteins, but beyond that it is also deemed as a prerequisite for the other functional properties [29]. The solubility of BBPI, BBPI-G and UBBPI-G conjugates were shown in Figure 4. It could be observed that the solubility of BBPI-G and UBBPI-G was slightly higher than the native BBPI. The solubility of BBPI was evidently enhanced by the combination with glucose despite the increase in the surface hydrophobicity (as shown in Figure 3). Normally, when hydrophobic groups are exposed, the protein molecules can be rearranged into larger supramacromolecular complexes by non-covalent interaction, resulting in a decrease of solubility. However, the increase of BBPI-G solubility might be owing to the attachment of a hydrophilic saccharide on the protein surface, and the hydrogen bonding capacity of saccharide’s –OH group could lead to an increased affinity between proteins and water molecules [29,36]. Furthermore, the attachment of glucose might also inhibit the non-covalent interaction of protein molecules to facilitate protein dissolution [37]. 

In addition, as shown in the DG study, the ultrasonic pretreatment could improve the reaction between protein and glucose. In fact, more hydrophilic glucoses conjugated to protein molecules via ultrasound pretreatment Maillard reaction than classical wet heating were found at the same reaction time. Therefore, the large amount of hydrophilic groups resulted in the increasing solubility of UBBPI-G. Meanwhile, ultrasonic treatment could make proteins unfolding and peptide bonds breaking, transforming insoluble protein aggregates into soluble ones [25]. Hence, ultrasonic assisted glycosylation was a more effective method in improving the solubility of protein.

### 3.6. Emulsifying Property

**Figure 5:**
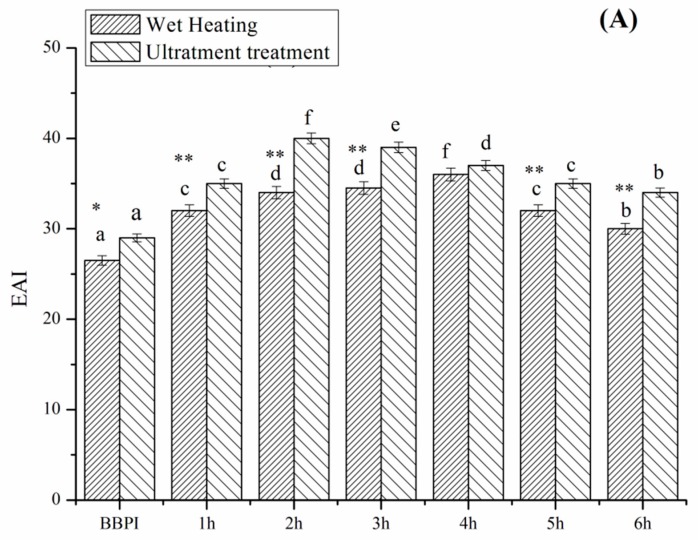

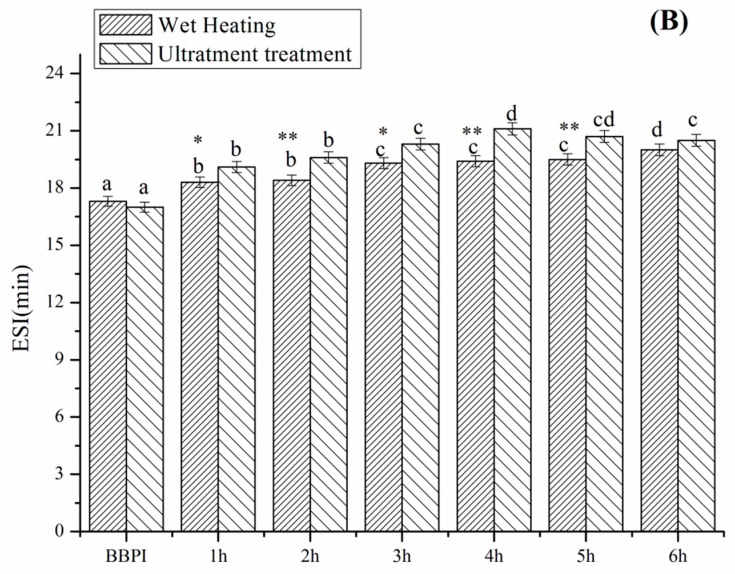
Emulsification activity (**A**) and emulsification stability (**B**) of BBPI by wet heating and ultrasound treatment Maillard reaction obtained after 1–6 h, respectively. Means with dissimilar lower-case letters indicate significant differences (*p* < 0.05), ** means in the same time *p* < 0.01, * means in the same time *p* < 0.05.

The emulsifying activity index (EAI) and emulsifying stability index (ESI) of grafted BBPI by classical heating and ultrasound pretreatment Maillard reaction at different reaction times is shown in Figure 5. The protein molecules have strong adsorbing ability on the oil–water interface and the saccharides dissolve well in the aqueous phase. Therefore, the protein-saccharide conjugates colligated the two characteristic properties of protein and saccharides often exhibit favourable emulsifying property [7]. As expected, the EAI and ESI of BBPI-G conjugates increase significantly (*p* < 0.05) after glycosylation. This result was the same as that obtained by Wang et al. [29] on the mung bean protein isolate (MBPI)-glucose (G) conjugate. Glycosylation optimized the hydrophobic-hydrophilic balance on the protein surfaces and modified the protein surface properties, which supported the emulsion stability through electrostatic interaction [38]. Furthermore, BBPI-G conjugates were predicted to show better emulsifying properties, due to the more unordered, less compacted and more flexible structure than native BBPI.

Compared with the BBPI-G conjugates, the UBBPI-G conjugates achieved higher EAI and ESI. These phenomena were consistent with the results reported by Zhang et al. [5], reporting that significant increase in EAI and ESI of β-conglycinin-maltodextrin samples was attributed to the exposure of the internal hydrophobic groups of protein under ultrasonic treatment, which reacted easily with the reducing-end carbonyl group in sugars and favoured emulsion formation and stabilization. Moreover, the ultrasound treatment was capable of accelerating the molecules mobility and the adsorption on the oil-water interfaces because of the mechanical effects caused by cavitation. Additionally, it can be observed that the trends of the emulsifying property as a function of reaction time were consistent with the solubility result (Figure 4). Therefore, the enhanced emulsifying property was probably correlated with the higher solubility, which demonstrated that the solubility was an important factor to assess the emulsifying ability [39]. 

### 3.7. Antioxidant Activity

**Figure 6:**
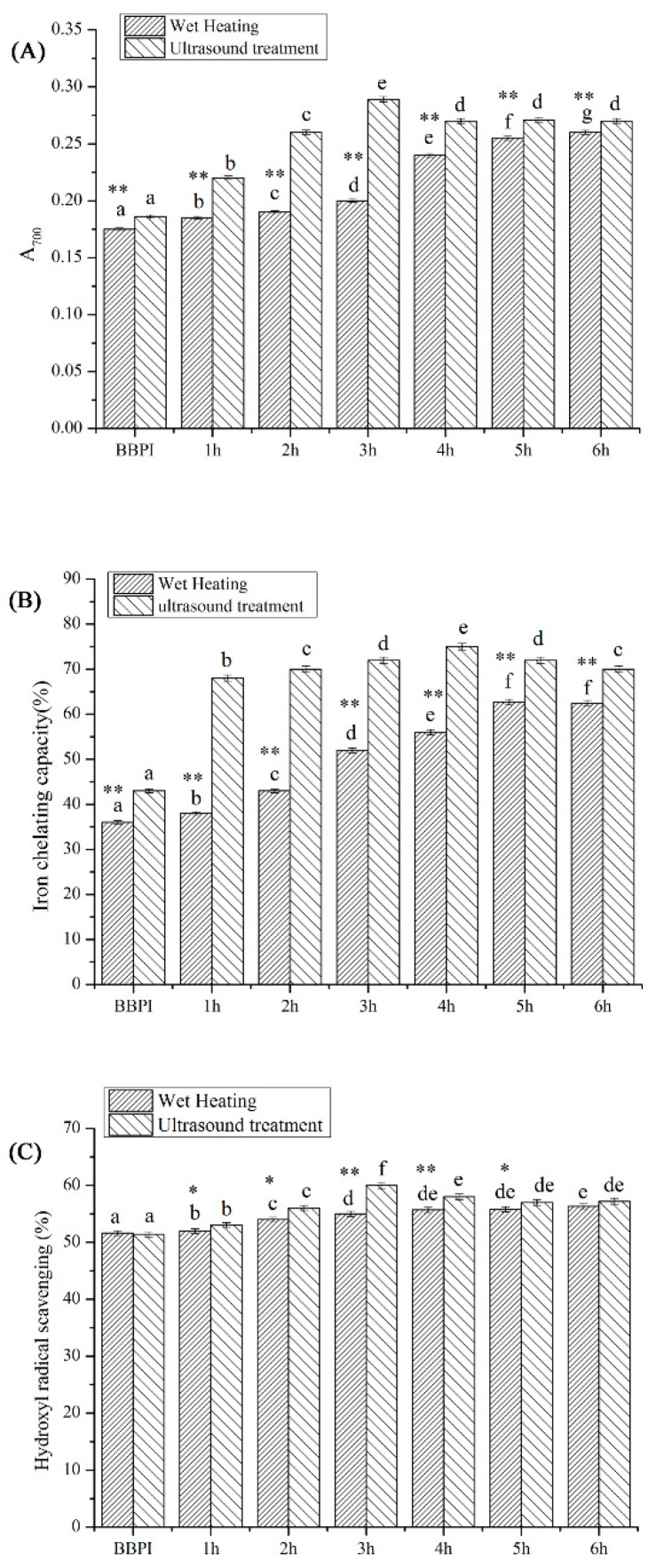
Antioxidant activity of BBPI by wet heating and ultrasound treatment Maillard reaction obtained after 1–6 h, respectively: (**A**) iron chelating capacity, (**B**) reducing power and (**C**) hydroxyl radical scavenging activity. Means with dissimilar lower-case letters indicate significant differences (*p* <0.05), ** means in the same time *p* < 0.01, * means in the same time *p* < 0.05.

The changes of BBPI-G antioxidant activity were shown in Figure 6. The iron chelating capacity of BBPI was improved after conjugation with glucose, which might be due to the formation of the high molecular weight compound prepared by the cross-linking of the free amino acids with sugars [40]. This result was same as that obtained by Liu et al. [41], who showed that the high molecular weight compound possessed strong iron chelating capacity. Furthermore, Liu et al. [42] predicted that the Maillard reaction was an effective method to improve the free radical scavenging, iron chelating activity and reducing power of proteins. Accordingly, the hydroxyl radical scavenging activity and reducing power of BBPI-G were both drastically developed after Maillard reaction. This phenomenon may be ascribed to the finding that the intermediate compounds of Maillard reaction were known as reductones, which exhibited good ability to break the radical chain as hydrogen atoms donator [8]. On the other hand, glycosylation could cause the structural changes in protein molecules, which could generate a wide range of compounds, leading to the preparation of conjugates that contributed to the reducing power [43].

Compared with the classical heating treatment, the ultrasound pretreatment Maillard reaction showed higher antioxidant potential, which was similar with the results obtained by Abdelhedi et al. [44]. They found that the conjugates with higher antioxidant ability could be prepared via ultrasound pretreated Maillard reaction instead of conventional process. This fact might be caused by the higher DG obtained by ultrasound treatment, which could produce more intermediate compounds, functioning as antioxidants. Similarly, Daglia et al. [45] explained that advanced Maillard reaction productions were the particular complex mix including numerous compounds. Hence, the protein antioxidant activity was improved by the contribution of the product mixture.

## 4. Conclusions

In this study, BBPI-G and UBBPI-G conjugates with higher molecular weight were successfully prepared, using the classic and ultrasound pretreated Maillard reaction. Compared with two types of glycosylation, we found that the Maillard reaction could be effectively promoted by ultrasound pretreatment to obtain higher DG in the same reaction time. This behaviour can be attributed to the finding that ultrasound could change the secondary and tertiary structure of BBPI, unfold the protein molecules, increase the speed of molecule motion, and then accelerate the Maillard reaction. In addition, UBBPI-G also exhibited significantly higher levels of the surface hydrophobicity, solubility, emulsification activity, emulsification stability and antioxidant activity than native BBPI and BBPI-G. Therefore, the combination of ultrasound pretreatment and Maillard reaction is expected to find applications in producing conjugates with desirable function properties.

## Figures and Tables

**Table 1:** DG and browning values of BBPI-G conjugates and UBBPI-G conjugates.

Time	BBPI-G		UBBPI-G	
	DG	Browning(A_420_)	DG	Browning(A_420_)
1 h	8.70 ± 0.19a **	0.155 ± 0.007a **	17.04 ± 0.12a	0.191 ± 0.005a
2 h	10.44 ± 0.42b **	0.179 ± 0.014ab*	20.49 ± 0.10b	0.212 ± 0.011a
3 h	14.94 ± 0.21c **	0.198 ± 0.012bc **	23.77 ± 0.16c	0.261 ± 0.011b
4 h	18.83 ± 0.03d **	0.221 ± 0.012c **	28.89 ± 0.31d	0.298 ± 0.015c
5 h	20.60 ± 0.38e **	0.248 ± 0.011d **	34.08 ± 0.21e	0.337 ± 0.021d
6 h	21.60 ± 0.27f **	0.293 ± 0.021e **	33.87 ± 0.14e	0.397 ± 0.011e

** means in the same time *p* < 0.01, * means in the same time *p* < 0.05. Different letters within the same column are statistically different (*p* < 0.05).

**Table 2:** Secondary structure distribution of BBPI by wet heating and ultrasound treatment Maillard reaction for 1–6 h.

Sample	α-helix (%)	β-sheet (%)	β-turn (%)	Random Coil (%)
BBPI	16.86 ± 0.11e	35.65 ± 0.29e	32.22 ± 0.20a	15.27 ± 0.03a
BBPI-G (1 h)	16.26 ± 0.11d	35.11 ± 0.10cde	33.30 ± 0.21b	15.33 ± 0.12a
BBPI-G (2 h)	15.78 ± 0.24c	35.35 ± 0.19de	33.34 ± 0.22b	15.53 ± 0.30ab
BBPI-G (3 h)	15.65 ± 0.21bc	35.31 ± 0.24de	33.13 ± 0.17b	15.91 ± 0.20bc
BBPI-G (4 h)	15.41 ± 0.21abc	35.32 ± 0.18de	33.07 ± 0.41b	16.20 ± 0.51cd
BBPI-G (5 h)	15.20 ± 0.13ab	35.09 ± 0.21cde	33.23 ± 0.18b	16.48 ± 0.31de
BBPI-G (6 h)	15.18 ± 0.15ab	34.33 ± 0.15b	34.02 ± 0.31cd	16.47 ± 0.35de
UBBPI	16.47 ± 0.22de	35.01 ± 0.39cd	33.26 ± 0.44b	15.26 ± 0.21a
UBBPI-G (1 h)	15.64 ± 0.27bc	34.93 ± 0.35cd	33.50 ± 0.32bc	15.93 ± 0.22bc
UBBPI-G (2 h)	15.23 ± 0.11ab	34.58 ± 0.44bc	34.14 ± 0.27d	16.05 ± 0.37bcd
UBBPI-G (3 h)	15.11 ± 0.17a	34.12 ± 0.23ab	34.52 ± 0.26d	16.25 ± 0.31cd
UBBPI-G (4 h)	15.02 ± 0.27abc	33.59 ± 0.17a	34.53 ± 0.31d	16.86 ± 0.13e
UBBPI-G (5 h)	15.09 ± 0.44a	34.01 ± 0.22ab	33.13 ± 0.27b	17.77 ± 0.17f
UBBPI-G (6 h)	15.08 ± 0.16a	34.04 ± 0.28ab	33.05 ± 0.33a	17.83 ± 0.21f

Different letters within the same column are statistically different (*p* < 0.05).
